# Association of behavioral health factors and social determinants of health with high and persistently high healthcare costs

**DOI:** 10.1016/j.pmedr.2018.06.017

**Published:** 2018-06-27

**Authors:** Stacy Sterling, Felicia Chi, Constance Weisner, Richard Grant, Alix Pruzansky, Sandy Bui, Philip Madvig, Robert Pearl

**Affiliations:** a Division of Research, Kaiser Permanente Northern California, United States; b University of California, San Francisco, United States; c The Permanente Medical Group, United States

**Keywords:** Social determinants of health, Behavioral health, High utilizer, Cost, ACEs

## Abstract

A high proportion of U.S. health care costs are attributable to a relatively small proportion of patients. Understanding behavioral and social factors that predict initial and persistent high costs for these “high utilizers” is critical for health policy-makers. This prospective observational study was conducted at Kaiser Permanente Northern California (KPNC), an integrated healthcare delivery system with 4.1 million members. A stratified random sample of high-cost vs. non-high-cost adult KPNC members matched by age, gender, race/ethnicity, type of health insurance, and medical severity (*N* = 378) was interviewed between 3/14/2013 and 3/20/2014. Data on health care costs and clinical diagnoses between 1/1/2008 and 12/31/2012 were derived from the electronic health record (EHR). Social-economic status, depression symptoms, adverse childhood experiences (ACEs), interpersonal violence, financial stressors, neighborhood environment, transportation access, and patient activation and engagement were obtained through telephone interviews. Initial and subsequent high-cost status were defined as being classified in top 20% cost levels over 1/1/2009–12/31/2011 and 1/1/2012–12/31/2012, respectively. Psychiatric diagnosis (OR 2.55, 95% CI 1.52–4.29, *p* < 0.001), financial stressors (OR 1.97, 95% CI 1.19–3.26, *p* = 0.009), and ACEs (OR 1.10, 95% CI 1.00–1.20, *p* = 0.051) predicted initial high-cost status. ACEs alone predicted persistent high-cost status in the subsequent year (OR 1.12, 95% CI 1.00–1.25, *p* = 0.050). Non-medical factors such as psychiatric problems, financial stressors and adverse childhood experiences contribute significantly to the likelihood of high medical utilization and cost. Efforts to predict and reduce high utilization must include measuring and potentially addressing these factors.

## 1 Introduction

Understanding the factors that drive healthcare utilization by the nation's costliest healthcare consumers is a top priority of health policy-makers. Most U.S. healthcare costs are attributable to a strikingly small percentage of “high utilizer” patients. Recent healthcare expenditure data suggest that 1% of patients account for 23% of total healthcare expenditures, 5% for almost half, and the bottom 50% for only 3%. For many individuals, high utilization persists; a fifth of the top 1% and almost half of the top 10% in 2009 health expenditures retained that status in 2010 (National Institute for Health Care Management Foundation, 2012).

High utilizers are typically sicker than low utilizers, and they differ from the general population in demographic characteristics and insurance coverage (Cohen and Yu, 2012). Separating their need for specific medical services from service use that is driven by other social and behavioral factors is challenging but critical. Social and behavioral factors have been shown to affect health and healthcare utilization, (Anderson et al., 2010; Boscardin et al., 2015; Brook et al., 2006; Chechulin et al., 2014; Hunter et al., 2015; Leininger et al., 2015; Maeng et al., 2015; Gawande, 2011) but few studies have systematically examined their association with healthcare costs, independent of medical condition severity.

### 1.1 Comorbid psychiatric and substance use conditions

Behavioral health problems frequently co-occur with medical conditions, and may cause, result from, and/or exacerbate them (Boscarino et al., 2012; Gunn et al., 2012). The literature suggests psychiatric disorders are strongly associated with high utilization of the emergency department (Ford et al., 2004), inpatient services, and sometimes, although not always, higher costs (Borckardt et al., 2011). Patients with substance use disorders in specialty treatment have higher prevalence of numerous health problems and may underuse primary care (Institute of Medicine, 2006; Mclellan and Woodworth, 2014; Mertens et al., 2003), while overusing emergency services (Cederbaum et al., 2014; Parthasarathy et al., 2012; Smith et al., 2015).

### 1.2 Adverse Childhood Experiences (ACEs) and Interpersonal Violence (IPV)

Exposure to stressful, traumatic events as a child has a well-established association with serious health conditions (Parthasarathy et al., 2012; Anda et al., 2008; Anda et al., 2002; Anda et al., 1999; Anda et al., 2009; Anda et al., 2001; Aydin et al., 1995; Dube et al., 2001; Dube et al., 2002a; Dube et al., 2009; Felitti, 1991; Felitti, 1993; Felitti, 2002). ACEs are common, with a dose-response relationship between number of ACEs experienced and severity of health problems (Campbell et al., 2016). Many studies have examined the neurobiological and physiological sequelae of early traumatic experiences (De Bellis and Thomas, 2003; Gorman et al., 2002; Gutman and Nemeroff, 2002; Larkin et al., 2014; Zanchi et al., 2012; Anda, 2008), but few have examined the relationship between ACEs and healthcare utilization (Anda et al., 2008; Felitti, 1991; Anda et al., 2007; Cannon et al., 2010; Mercado et al., 2015), and, to our knowledge, none have examined the independent relationship between ACEs and healthcare costs, controlling for other factors including medical conditions and severity. Interpersonal violence (IPV) is also associated with health and mental health conditions, in addition to direct medical sequelae from violence, e.g., injuries, and several studies have documented the association between IPV and higher healthcare utilization and cost (Brown et al., 2008; Corso et al., 2007; Bonomi et al., 2009).

### 1.3 Patient Activation and Engagement

Patient activation (knowledge, resources and self-efficacy regarding health conditions, healthcare system interactions and self-management), and patient engagement (ability to engage in behaviors to enhance health and well-being and prevent or inhibit disease) (Institute of Medicine, 2001; Bernabeo and Holmboe, 2013) are central to patient-centered models of care recommended by the Institute of Medicine (Institute of Medicine, 2001). They enable patients to “communicate effectively with healthcare providers, practicing health-related problem solving, and decision-making.” Low activation is associated with worse patient outcomes (Hibbard et al., 2013; Hibbard and Greene, 2013) and higher costs (Hibbard et al., 2013).

### 1.4 Financial stressors and lifestyle factors

The literature is mixed on whether financial stressors affect utilization and costs, acting as barriers to seeking care, using preventive healthcare, or adhering to medication, thus causing emergency and inpatient care later on (Banthin and Bernard, 2006; Piette and Heisler, 2004). Lifestyle factors such as smoking, exercise and obesity may also affect healthcare costs given their close association with health and well-being (Kvaavik et al., 2010).

Many studies using quantitative methods/algorithms for identifying and predicting utilization status rely solely on insurance claims and diagnostic data, an approach hampered by its reliance on limited clinical data, lack of sensitivity, and the delay from service delivery to data availability (Health Care Transformation Task Force, 2015). This study combines administrative, electronic health record (EHR) and self-report data to address the question: “What behavioral health and social factors are associated with initial and persistent high cost status?”, by examining an array of behavioral and social factors (mental health conditions and substance use disorders, ACEs, IPV, socioeconomic status (SES), financial stressors, lifestyle factors, and patient activation and engagement) among a sample of high utilizers and non-high utilizers matched on demographic characteristics, medical severity and insurance type.

## 2 Methods

### 2.1 Setting

The study site was Kaiser Permanente Northern California (KPNC), a large, non-profit, group model managed care integrated health delivery system, covering 4.1 million members, 31% of the region's population. Of the membership, 45% are commercially insured, 28% insured through Medicare and 9% Medicaid, and 18% through other coverage.

### 2.2 Sample for telephone interview

From a dataset of 1,721,646 adult KPNC members with continuous membership during 1/1/2009–12/31/2011, we excluded healthy members and severely frail or near end-of-life members, and focused on those with either a well-managed chronic condition, or a significant but stable disability (including mental disability) [segment A], and those with multiple chronic conditions, including mental health disorders (or diagnoses) or advanced illness [segment B] (*N* = 915,535) (Abrams et al., 2016).

We stratified the 915,535 patients by health plan product line (Commercial, Medicare Special Needs Plans (SNP), Medicare Non-SNP, Medicaid Seniors & Persons with Disabilities (SPD), and Medicaid Non-SPD) and by medical morbidity group (segment A or B), resulting in 10 unique strata. From each, we randomly selected equal numbers of “high-cost patients” (classified in the top 20% ranked by dollars spent over 1/1/2009–12/31/2011) and “non-high-cost patient” matched by age (exactly or within 5 years), gender, race/ethnicity (African American, Asian Pacific Islander, Hispanic, Native American, White) and segment-specific DxCG risk score quartile. DxCG prospective relative risk scores are validated measures of overall disease burden (Verisk Health Inc. Sightlines DxCG Risk Solutions, 2010) (Wagner et al., 2016). We matched on segment-specific DxCG risk score quartile and segment number as a proxy measure of aggregate disease severity and service need, allowing us to examine the behavioral health and social factors that distinguish persistent high cost patients from their counterparts with similar burden of medical disease.

Chart reviews excluded members with rare, anomalous, or exceedingly costly conditions (e.g., catastrophic complications or severe trauma). New subjects were resampled after all potential subjects from the original sampling frame were interviewed. If one or both of the matched pair was further determined as ineligible (e.g., unable to be located, too ill), a replacement pair was resampled until we reached the goal of 20 completed pairs per stratum or the end of the study. A total of 405 interviews were completed out of 521 eligible patients (RR = 78%); for 27 out of the 405 completed interviews, the interview of the matching counterpart was not completed, leaving an analytical sample of 189 high-cost patients and 189 matched non-high-cost counterparts (*N* = 378).

### 2.3 Measures

Telephone interviews collected data on: SES (income, employment, education); living situation, legal and conservatorship status; access to mobile or electronic healthcare (including patient portal); Patient Activation Measure (PAM) scale (Hibbard et al., 2005); depression symptoms measured by the Patient Health Questionniare-9 Item (PHQ-9) (Kroenke et al., 2001); smoking and alcohol and drug use. We used validated instruments to measure ACEs (physical, sexual or emotional abuse, physical or emotional neglect, having a substance abusing or mentally ill parent, having an incarcerated parent, and witnessing domestic violence); IPV (adult experiences of physical, emotional or financial abuse by intimate partner); financial stressors (whether member needed to: borrow money, cut back on necessities or delay care due to medical expenses); neighborhood environment (crime and safety, physical and social disorder) and transportation access (having a car to use, driver's license, primary mode of transportation).

We identified EHR-based ICD-9 diagnoses for psychiatric disorders, alcohol, drug and tobacco use disorders, chronic and other conditions between 1/1/2008 and 12/31/2011, along with obesity diagnoses, body mass index (BMI) values and exercise patterns for each subject. Utilization and costs by type of services were extracted from administrative databases and estimated for each participant.

### 2.4 Statistical analyses

We compared differences in all measures between high-cost and non-high-cost patients using chi-square or Fisher Exact tests for categorical variables and *t*-tests for continuous variables. All behavioral health and social determinant factors significant at *p* < 0.10 level were considered candidate predictors for multivariable modeling. We examined two outcomes, initial high-cost status (classified in top 20% cost levels over 1/1/2009–12/31/2011) and subsequent high-cost status (in the top 20% cost levels in year 2012) and conducted stepwise logistic regressions to construct efficient models of medical, behavioral health and social factors associated with each. In these models, all the matching variables and four medical conditions prevalence rates of which were significantly different between the two groups after matching (asthma, arthritis, chronic obstructive pulmonary disease (COPD), and Chronic Pain) were forced to enter the model, followed by other variables that were entered and retained if *p* ≤ 0.1. All analyses were conducted using SAS 9.3.

## 3 Results

After matching, we found no differences in age, gender and racial/ethnic distributions between high-cost and non-high-cost patients. The mean age was 64; 75% were female; 61% were White. There were no differences in education, employment status, income, or living conditions. Higher proportions of high-cost patients were separated, divorced or never married (51% vs. 37%, *p* = 0.029).

The two groups were not different in prevalence of 12 of the 16 chronic conditions examined, including: diabetes, hypertension, congestive heart failure, ischemic heart disease, osteoporosis, epilepsy and cancer. However, we found higher prevalence among high-cost patients of four conditions: asthma, arthritis, COPD and chronic pain (37% vs. 27%, *p* = 0.048; 78% vs. 65%, *p* = 0.003; 32% vs. 18%, *p* = 0.001 and 42% vs. 29%, *p* = 0.007, respectively). In addition, high-cost patients had a higher overall disease burden than their matched non-high-cost counterparts: 70% had 3 or more chronic conditions compared to 59% of non-high-cost patients (*p* = 0.040), and 70% had both physical and behavior health conditions compared to 53% of non-high-cost patients (*p* = 0.001) (not shown).

### 3.1 Behavioral health conditions

High-cost patients had higher prevalence of EHR-recorded anxiety, depression, major psychosis, personality disorders and other psychiatric conditions (Table 1). Self-report of mood symptoms in the structured interviews confirmed that high-cost patients had higher PHQ-9 scores and greater functional impairment and service need. Prevalence of substance use disorders did not differ between the groups (Table 2).

**Table 1:** Comparisons of behavioral health conditions, behavioral and social factors, high-cost patients versus non-high-cost patients.

	High-cost patients (*N* = 189)	Non-high-cost patients (*N* = 189)	*p* Value
Behavioral health conditions derived from electronic health record (EHR)			
Psychiatric diagnoses in EHR, 2008–2011			
Anxiety disorders, *N* (%)	99 (52.4%)	57 (30.2%)	<0.001
Depression, *N* (%)	104 (55.0%)	73 (38.6%)	0.001
Major psychosis, *N* (%)	37 (19.6%)	15 (7.9%)	0.001
Personality disorders, *N* (%)	16 (8.5%)	2 (1.1%)	<0.001
Other psychiatric conditions, *N* (%)	61 (32.3%)	22 (11.6%)	<0.001
Any psychiatric conditions, *N* (%)	137 (72.5%)	99 (52.4%)	<0.001

Substance use diagnoses in EHR, 2008–2011			
Alcohol psychosis, *N* (%)	2 (1.1%)	3 (1.6%)	NS
Alcohol abuse, *N* (%)	9 (4.8%)	9 (4.8%)	NS
Alcohol dependence, *N* (%)	10 (5.3%)	8 (4.2%)	NS
Drug psychosis, *N* (%)	8 (4.2%)	2 (1.1%)	0.055
Drug abuse, *N* (%)	22 (11.6%)	14 (7.4%)	NS
Drug dependence, *N* (%)	18 (9.5%)	11 (5.8%)	NS
Any substance use disorders (excluding tobacco dependence), *N* (%)	36 (19.1%)	27 (14.3%)	NS
Behavioral and social factors derived from telephone interviews			
PHQ-9 (past 2 weeks)			
Mean (SD)	6.7 (6.3)	4.9 (5.3)	0.004
Severity/treatment need, *N* (%)			
PHQ-9 ≤ 4 (no treatment need)	91 (48.2%)	106 (56.1%)	0.043
5 ≤ PHQ-9 ≤ 14 (physician judgement)	72 (38.1%)	71 (37.6%)	
PHQ-9 ≥ 15 (warrant treatment)	26 (13.8%)	12 (6.4%)	
Major depression syndrome, *N* (%)	28 (14.8%)	15 (7.9%)	0.035
Minor depression syndrome, *N* (%)	27 (14.3%)	8 (4.2%)	0.001
Function impaired, *N* (%)	35 (18.5%)	18 (9.5%)	0.012

Adverse Childhood Experiences (ACEs)			
Total ACE score, mean (SD)	2.8 (2.8)	1.9 (2.4)	0.001
Childhood abuse by category			
Emotional, *N* (%)	70 (37.4%)	41 (21.9%)	0.001
Physical, *N* (%)	60 (32.3%)	33 (17.6%)	0.001
Sexual, *N* (%)	40 (21.7%)	31 (16.6%)	NS
Neglect by category			
Emotional, *N* (%)	67 (36.0%)	50 (26.6%)	0.049
Physical, *N* (%)	25 (13.4%)	18 (9.6%)	NS
Household dysfunction by category			
Substance abuse, *N* (%)	66 (35.3%)	49 (26.1%)	0.053
Mental illness, *N* (%)	46 (25.0%)	36 (19.3%)	NS
Mother treated violently, *N* (%)	44 (23.9%)	20 (10.9%)	0.001
Incarcerated household member, *N* (%)	27 (14.4%)	15 (8.1%)	0.052
Parental separation or divorce, *N* (%)	77 (42.1%)	68 (36.6%)	NS

Interpersonal violence (IPV)			
Have been abused emotionally, physically or financially, *N* (%)	88 (46.6%)	67 (35.5%)	0.028

Financial stressors: had to do the following because of the amount that you had to pay for medical care, *N* (%)			
Borrow money	35 (18.5%)	13 (6.9%)	<0.001
Cut back on/go without some necessity	37 (19.6%)	22 (11.6%)	0.034
Delay/avoid ER/office visits	32 (16.9%)	19 (10.1%)	0.050
Use medication less often than prescribed	23 (12.7%)	10 (5.7%)	0.023
Switch to A cheaper medication	31 (17.2%)	18 (10.3%)	0.061

Patient activation			
Total PAM score, mean (SD)	65.0 (17.4)	66.5 (17.4)	NS
Patient activation level, *N* (%)			0.006
Level 1: starting to take a role (PAM ≤ 45.2)	31 (16.4%)	14 (7.4%)	
Level 2: building confidence/knowledge (47.4 ≤ PAM ≤ 52.9)	24 (12.7%)	44 (23.3%)	
Level 3: taking action (56.4 ≤ PAM ≤ 66.0)	53 (28.0%)	50 (26.5%)	
Level 4: maintaining behaviors (PAM ≥ 68.5)	81 (42.9%)	81 (42.9%)	

Notes: PHQ-9 = patient health questionnaire. PAM = patient activation measures. SD = standard deviation. NS = non-significant. *p* Values from Chi-square or Fisher's Exact Tests.

**Table 2:** Logistic regression models with stepwise selection of behavioral health and social factors independently associated with high cost status in 2009–2011 and 2012.

Set 1. Outcome = high cost status in 2009–2011						
	Step 1⁎		Step 2⁎		Step 3⁎	
	OR (95% CI)	*p* Value	OR (95% CI)	*p* Value	OR (95% CI)	*p* Value
Any psychiatric diagnosis (yes vs. no)	2.60 (1.56, 4.31)	<0.001	2.65 (1.58, 4.44)	<0.001	2.55 (1.52, 4.29)	<0.001
Any financial burden (yes vs. no)	–	–	2.21 (1.35, 3.61)	0.002	1.97 (1.19, 3.26)	0.009
ACE (per 1 unit increase)	–	–	–	–	1.10 (1.00, 1.20)	0.051

Set 2. Outcome = high cost status in 2012						
	Step 0⁎⁎		Step 1⁎⁎			
	OR (95% CI)	*p* Value	OR (95% CI)	*p* Value		
High cost status in 2009–2011 (yes vs. no)	4.46 (2.61, 7.60)	<0.001	4.19 (2.45, 7.19)	<0.001		
ACE (per 1 unit increase)	–	–	1.12 (1.00, 1.25)	0.050		

⁎ Models adjusted for age, gender, white race, product line, segment, DxCG quartile, having a diagnosis of asthma, arthritis, COPD, and chronic pain in 2008–2011. Potential behavioral health and social factors to select from include having any psychiatric diagnosis in 2008–2011, patient activation measures (PAM) level (level 2–4 vs level 1), adverse childhood experiences (ACE), any interpersonal violence (IPV), and any financial burden. We did not include Patient Health Questionnaire (PHQ-9) in the model because the measure was highly correlated with having any psychiatric diagnosis. NS = Non-significant. OR = Odds Ratio. 95% CI = 95% Confidence Interval.

⁎⁎ Models adjusted for age, gender, white race, product line, segment, DxCG quartile, having a diagnosis of asthma, arthritis, COPD, and chronic pain in 2008–2011. High cost status in 2009–2011 was forced entered in the modeling steps. Potential behavioral health and social factors to select from include having any psychiatric diagnosis in 2008–2011, patient activation measures (PAM) level (level 2–4 vs level 1), adverse childhood experiences (ACE), any interpersonal violence (IPV), and any financial burden. We did not include Patient Health Questionnaire (PHQ-9) in the model because the measure was highly correlated with having any psychiatric diagnosis. Step 0: Initial model with only high cost status in 2009–2011 and control variables. Step 1: Behavioral health and social factors were added to the model, among them only ACE was entered and retained. NS = Non-significant. OR = Odds Ratio. 95% CI = 95% Confidence Interval.

### 3.2 Other social and behavioral factors

High-cost patients had significantly higher ACE scores than the matched non-high-cost patients, and higher rates of several specific ACEs exposures. Significantly higher proportions of high-cost patients reported each of the medical care-related financial burdens (Table 2). Average PAM scores did not differ between the groups (Table 2). We found no differences in access to computer or email, cell phone ownership, or patterns of portal use between groups. We found no differences between groups in perceptions of issues or concerns related to neighborhood environment, or in self-reported past or current smoking or diagnosed tobacco dependence. No significant differences were found in either the BMI categories or in obesity diagnoses, or in self-reported exercise, in minutes per day, days per week, or total amount per week (not shown).

### 3.3 Multivariable logistic regression models

In the multivariable logistic regression analysis including those behavioral and social factors found to be associated with initial high-cost status in 2009–2011 (*p* < 0.10), we excluded PHQ-9 because of multicollinearity with the indicator of having any psychiatric diagnosis. Results suggested that having a psychiatric diagnosis (OR 2.55, 95% CI 1.52–4.29, *p* < 0.001), having financial burden (OR 1.97, 95% CI 1.19–3.26, *p* = 0.009), and ACE score (OR 1.10, 95% CI 1.00–1.20, *p* = 0.051) were independently associated with high-cost status in 2009–2011 (Table 2). Similar analyses were conducted to examine factors associated with high-cost status in year 2012 while adjusting for demographics, product line, severity, and high-cost status in 2009–2011. Results suggested that high-cost patients in 2009–2011 were >4 times more likely than others to be high-cost patients in 2012 (OR 4.19, 95%CI 2.45–7.19, *p* < 0.001). Among the behavioral health and social determinants factors examined, ACE was the only significant factor associated with high-cost status in 2012 (OR 1.12, 95% CI 1.00–1.25, *p* = 0.050) (Table 2).

## 4 Discussion

Several behavioral health and social factors were found to be independently associated with high-cost status. Our finding that a psychiatric diagnosis is associated with initial high-cost status is consistent with studies suggesting that many high utilizers struggle with the burden of mental health problems which can influence healthcare decisions in significant and costly ways (Institute of Medicine, 2006). The co-occurrence of mental health and medical conditions can prolong and complicate treatments (Moussavi et al., 2007) and amplify people's experiences of symptoms (e.g., chronic pain may be perceived as more severe), which could conceivably drive patients to seek potentially costly and unneeded or avoidable urgent or emergency care (Arnow et al., 2009). Mental health conditions may also compromise patients' ability to cope with medical conditions, navigate the healthcare system (Katon et al., 2010), and engage in appropriate self-care (Morris et al., 2011). Mental health interventions which help improve symptoms in patients with anxiety and depression could ultimately lead to better overall health status, and/or reduced utilization of potentially avoidable and often costly healthcare services such as ED and inpatient care. Increasing integration of such interventions into primary care and other medical settings (Thota et al., 2012), and medical care into specialty psychiatry settings (Weisner et al., 2001), can improve quality of care and potentially curtail unnecessary utilization and costs (Jacob et al., 2012).

Although carefully matched on medical severity, there remained some differences, and higher disease burden and the presence of three or more co-occurring medical conditions were related to high cost status. Greater medical complexity often results in the need for more healthcare services, resulting in higher costs. Self-reported financial stressors are associated with initial high-cost status, though not persistent high-costs (Gordon, 2013). While poorer health is related to SES (Feinstein, 1993), healthcare-related costs have not necessarily been similarly associated. Healthcare providers might consider how financial stressors can affect utilization, and explore services linking members to resources such as food and financial assistance, and explore modifying prescription and other co-pay design, to facilitate the optimal use of healthcare.

To our knowledge, no other studies have examined the effects of ACEs on utilization and cost, independent of medical condition acuity. ACEs exposure emerged as the sole factor independently associated with both initial and persistent high-cost status, aside from initial high cost status. That ACE exposure continues to affect health services utilization and costs well into mid- and later-life (the mean age of both groups was 64) is striking and provides further evidence of the persistent impacts of childhood trauma exposure. The relationship between ACEs and health outcomes is complex, and likely involves both direct physiological impacts resulting from prolonged exposure to stress (Miller et al., 2011; Danese and BS, 2012; Chrousos et al., 1993), as well as indirect effects through mediating behaviors such as smoking and other substance use, sub-optimal nutrition and exercise activity and ongoing stress (Chung et al., 2010; Dube et al., 2002b; Felitti et al., 1998). It is plausible that in many cases, people adopt risky (but soothing) behaviors as a way of coping with ACEs exposure. These behaviors, along with ongoing environmental stressors, can lead to a host of health problems (e.g., smoking contributing to the development of COPD, poor nutrition and lack of exercise contributing to obesity and diabetes, etc.). These in turn may lead to increased healthcare utilization, and ultimately, higher costs. We found, however, that ACEs were independently predictive of high, and persistently high cost status (and were in fact the only behavioral or social factor which predicted persistent high cost status), controlling for medical severity and psychiatric conditions. This finding suggests that there are other mechanisms involved in the relationship between ACEs and healthcare costs. Additional research should further examine the role and mechanisms of childhood stress and trauma exposures as independent predictors of healthcare utilization, potentially identifying factors suitable for intervention. Even in the absence of a full understanding of the underlying mechanisms driving the association of ACEs with healthcare costs, our finding of this independent relationship between ACEs and high costs suggest the potential value of better and earlier ACEs screening, prevention, assessment and trauma-informed treatment in healthcare settings (Young-Wolff et al., 2016). Experiences of IPV as an adult, while not an independent predictor, was associated with high cost status at the bivariate level, underscoring the relationships we found between trauma exposure and healthcare costs.

Alcohol or drug problems did not emerge as predictors of high or persistently high cost status, nor did a number of other medical conditions, including diabetes, hypertension, congestive heart failure, ischemic heart disease, osteoporosis, epilepsy and cancer, likely due to the similar prevalence of these conditions in the two samples.

This study has several limitations. As with other observational studies, our results cannot be interpreted as causal. We found low rates of missingness for most measures in the analytical sample and did not perform multiple imputation or sensitivity analyses for missing data. While study results were based on comparisons between high cost and non-high cost patients matched on patient characteristics including age and gender, post hoc analyses found that among the eligible patients, those who had a complete interview were more likely to be younger or female, thus findings might be less generalizable to older male patients. The sample was drawn from a private, non-profit health system, thus findings may not be generalizable to public populations. However, the inclusion of Medicare (SNP and non-SNP) and Medicaid (SPD and non-SPD) beneficiaries increases its generalizability. A strength of this study is that we combined administrative, clinical EHR and self-report data to examine relationships between a number of social and behavioral factors and high utilizer status, while controlling for patient characteristics known to be associated healthcare utilization and cost.

### 4.1 Conclusion

Numerous efforts are underway in healthcare organizations across the nation to improve quality and reduce costly, avoidable health services utilization, many of which have focused on linking patients to community services such as transportation or case management. However, another approach may involve the use of modern technology, which is in many ways easier to implement. Ideally, health systems could offer patients a menu of behavioral health services, from low-intensity, self-guided options, including technological innovations such as online assessments and video visits and virtual groups, through intensive specialty treatment. Our findings underscore the importance of considering behavioral health and social factors in models predicting healthcare costs and utilization for use in the policy sphere (e.g., “Impactibility Models”) (Lewis, 2010), in services planning and case management, and in future research on healthcare costs and utilization. Future studies should examine more closely the role of behavioral and social factors on healthcare utilization and costs in specific populations, for example, the impact of co-occurring anxiety or depression specifically among patients with diabetes. As healthcare delivery systems increasingly adopt population-level disease management programs, information about the interplay between specific diseases and such factors, as they impact patient utilization behavior, could help inform programmatic and policy decisions. For healthcare systems that wish to curb unnecessary utilization and costs while improving the care of their complex patients, these findings provide further evidence that they may need to look beyond simply providing medical care, towards services which address patients' broader social and behavioral health needs.

## Conflicts of interest

None.
